# On-Time Appointment Keeping and Associated Factors among Human Immunodeficiency Virus-Positive Adult Patients Accessing Antiretroviral Therapy at Health Centers in East Gojjam Zone, Northwest Ethiopia, 2019

**DOI:** 10.1155/2023/1416187

**Published:** 2023-11-30

**Authors:** Mengistie Kassahun Tariku, Abebe Habtamu Belete, Daniel Tarekegn Worede, Sewnet Wongiel Misikir

**Affiliations:** ^1^Department of Public Health, College of Health Science, Debre Markos University, Debre Markos, Ethiopia; ^2^Department of Medical Laboratory Technology, Felege Hiwot Comprehensive Specialized Hospital, Bahir Dar, Ethiopia

## Abstract

**Background:**

The magnitude of on-time appointment keeping among HIV-positive adult patients was not identified in Ethiopia. Hence, this study aimed to assess on-time appointment keeping and associated factors among human immunodeficiency virus-positive patients accessing antiretroviral therapy in the East Gojjam Zone, Northwest Ethiopia.

**Methods:**

A community-based cross-sectional study was performed on 830 HIV-positive patients from April 1 to May 10, 2019, in East Gojjam Zone. A systematic random sampling technique was used to include study subjects, and data were collected through face-to-face interviews. Bivariable and multivariable binary logistic regression analyses were performed. Independent variables with a *P* value of <0.05 were considered statistically significant cut points.

**Results:**

The prevalence of on-time appointment keeping was 62.1%. Being >24 years old (adjusted odds ratio (AOR) = 2.13; 95% confidence interval (CI) = 1.54–4.25), being unmarried (AOR = 0.59; 95% CI = 0.45–0.82), taking a drug regimen of tenofovir + lamivudine (3TC) + efavirenz (EFV) (AOR = 2.11; 95% CI = 1.84–3.62), taking ART ≥12 months (AOR = 4.32; 95% CI = 2.22–8.40), having a mobile (AOR = 2.22; 95% CI = 1.44–3.64), and getting adherence support (AOR = 1.83; 95% CI = 1.16; 95% 1.16–3.50) were significant factors.

**Conclusion:**

On-time appointment keeping was low. Adherence support and appointment reminders should be strengthened.

## 1. Background

Adherence to antiretroviral therapy (ART) is an inclusive idea consisting of two basic components: medication adherence and on-time attendance at scheduled clinic appointments [1]. Appointments are classified as “on-time” if the patients attend the clinic within seven days of the scheduled clinic appointment [2]. On-time appointment keeping provides a high-level assessment of how well populations of patients at a clinic perform in picking up prescribed ART on or before the pill run-out date. It also estimates clinic performance in successfully engaging patients to attend scheduled appointments [2]. The World Health Organization (WHO) recommended that the acceptable clinic-level performance of on-time appointment keeping should be above 80% [2]. It is vital for 100% lifetime medication adherence [3].

In the management of human immunodeficiency virus (HIV) and acquired immune deficiency syndrome (AIDS), on-time clinical attendance for pharmacy refills, laboratory investigations, and clinician assessments are crucial for successful treatment achievements and good clinical outcomes [4]. When patients miss scheduled appointments, the opportunity to early identify and manage treatment failure vanishes [4, 5].

Although medication adherence is supposed to be the most important factor in treatment outcome, the previous study has revealed that missed clinic appointments from ART clinics are significantly associated with virological failure [6], drug resistance, and an increased risk of AIDS-related illness or death [5].

Even though there has been increased public and patient awareness of the ways of immunodeficiency virus (HIV) infection prevention and the benefits of antiretroviral therapy (ART) globally [7], the interruption of scheduled clinical appointments has been high in Africa [8]. Healthcare delivery systems, patient-related, sociodemographic, psychosocial, and clinical-related factors are associated with clinic appointment attendance [9, 10]. Sometimes HIV-positive patients are appointed orally. This leads to missing the appointment [5].

Antiretroviral therapy (ART) distribution and HIV/AIDS preventive programs are both localized in Ethiopia. As a result of prior research's primary focus on medication adherence, Ethiopia's current HIV treatment strategy is faith based [5], but there is limited evidence for the magnitude of on-time appointment keeping and associated factors [11]. Therefore, this study aimed to assess on-time appointment keeping and associated factors among human immunodeficiency virus-positive adult patients accessing antiretroviral therapy at health centers in East Gojjam Zone, Northwest Ethiopia.

## 2. Methods and Procedure

### 2.1. Study Design Setting

A community-based cross-sectional study design was performed from April 1 to May 10, 2019, at government health centers in East Gojjam Zone. This zone is found in the Amhara region, of Northwest Ethiopia. It is the second most populated zone in the region, with 2,719,119 total estimated population. There were eight government hospitals and 102 government health centers in this zone. Out of the total government health facilities, all hospitals and 23 health centers provided ART services and care for patients. About 11302 HIV-positive patients received ART at these health facilities [12]. All adult HIV-positive patients receiving ART and having scheduled appointments from May 03/2018 to May 03/2019, at selected government health centers in East Gojjam Zone were included in the study. In contrast, HIV-positive women who were pregnant, labouring, lactating women, or seriously ill patients were excluded. Those HIV-positive patients whose appointments had no registered appointment dates were also excluded.

### 2.2. Sample Size Determination and Sampling Technique

The sample size was determined using a single population formula. It was calculated via the formula (*n* = ((*Z*/2)^2^*P* (1 − *P*))/*S*^2^) with consideration of a 95% confidence interval and 5% margin error (*S*). The assumption of on-time appointment keeping of 43% was taken from the study conducted in three countries of Africa [5]. The calculated sample size was 377. This calculated sample size was also multiplied by the design effect of 2, and by adding 10% nonresponse, the total sample size was 830. A multistage sampling technique was employed to select study participants. The lottery method, a simple random sampling technique, was used to select 8 governmental health centers from 23 governmental health centers. The total sample size was proportionally distributed to eight governmental health centers. A total of 6139 eligible HIV-positive patients attended ART at selected health centers. During the time of the health center visit, study participants were selected by systematic random sampling at intervals of seven from each health center.

### 2.3. Data Collection Procedure

Based on a review of pertinent literature [13, 14], a structured questionnaire (Appendix 3) and checklist (Appendix 2) were designed. As identifiers, patient record numbers were used to connect patients with their medical histories. Primary data were gathered by HIV-positive community health workers who had completed grade ten or higher in education (HIV-positive voluntary community health workers are also employed as ART defaulter tracers in Ethiopia.) and conducted face-to-face interviews with patients. Patients' addresses were obtained from health centers. Home visits served as the method for gathering data. Secondary data were also taken from the medical records of chosen ART patients.

The dependent variable was on-time appointment keeping. Appointment keeping can be easily accessed from attendance records. If a patient has been visiting within seven days of all scheduled clinic appointments for 12 months, or within 24–48 hours before the “original” scheduled appointment [15], it is scored as 1 (yes); if a person has missed one or more scheduled clinic appointments for any reason(s), it is scored as 0 (no) [16]. This study did not consider unscheduled visits to an emergency room, an inpatient ward, or any ward [15].

Independent variables were sociodemographic (such as sex, age, residence, educational status, occupation, marital status, and monthly income), healthcare delivery systems (such as paper-based appointments, follow-up counseling, ever-faced unavailability of health workers, appointment time convenience, and satisfaction), patient-related, psychosocial (Appendix 3), and clinical-related factors (Appendix 2). The perceived social stigma was assessed using ten questions with “yes” or “no” response choices [17]. Perceived stigma was defined by having at least 1 yes answer out of the 10 questions. Satisfaction was measured by using sixteen questions [18]. Satisfaction was defined as having equal to median or above median of 16 satisfaction-related questions.

### 2.4. Data Quality Control

Before data collection, a questionnaire was translated into the local language (Amharic) and converted back into English to check its consistency. Two days of training were given to eight data collectors and four supervisors about the data collection process. To check the consistency of the questionnaire, a pretest was performed. The collected data were checked for completeness and consistency daily by supervisors. Before data entry, the completeness and consistency of the data were also checked.

### 2.5. Data Entry and Analysis

After checking data completeness, it was coded and entered into EpiData version 3.1, and then it was exported to SPSS version 23 for analysis. Before binary logistic regression analysis, descriptive analysis was performed. A bivariable analysis was conducted. Independent variables with a *P* value of <0.2 in bivariable binary logistic regression analysis were selected for multivariable binary logistic regression analysis. A multivariable binary logistic regression analysis was performed. Each independent variable with a *p* value of less than 0.05 was considered a statistically significant cut point in multivariable binary logistic regression analysis.

## 3. Results

### 3.1. Sociodemographic and Economic Characteristics

The study included 825 participants with a 99.4% response rate. Out of the total study participants, 416 (50.4%) were female. The median age was 37 years, with an interquartile range (IQR) of 33–43 years. Four hundred and sixty-nine (56.8%) were urban dwellers. Regarding educational level, 361 (43.2%) had no formal education. Nearly three-tenths (28.8%) of the participants were merchants. Four hundred and eighty-four (58.7%) were married. The median monthly income was 1720 (IQR = 1234 to 2562) birr (Table 1).

### 3.2. Clinical Characteristics

Among the total study participants, 369 (44.7%) had stage I HIV. The median CD4 count was 430 (IQR = 254–650) cells/mm^3^. The majority, 694 (84.1%) of participants, had a working functional status. Seven hundred and thirteen (86.4%) had normal nutritional status. More than half (475, or 57.6%) of the study participants have taken the TDF + 3TC + EFV drug regimen. Five hundred thirty-three (64.6%) people encountered an opportunistic infection. More than three-fourths, or 649 (78.7%), of participants, did not experience side effects (Table 2).

### 3.3. Psychosocial, Behavioral, and Healthcare Delivery System Characteristics

More than three-fifths of study participants had not faced stigma. Four hundred ninety-five (60.0%) were members of the PLWHA association. Three-fourths (74.2%) of the study participants had not received social support. Nearly, three-fifths, or 518 (62.8%) of study participants, have gotten paper-based appointments. Four hundred and seventy-eight (57.9%) had not gotten follow-up counseling. Nearly, three-fifths of study participants' appointment times were not convenient. Nearly half, that is, 389 (48.2%), were substance abusers (Table 3).

### 3.4. Outcome Data

**Figure 1:**
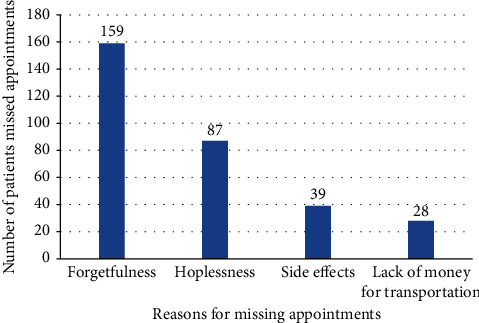
Reasons for missing appointments from ART clinic in East Gojjam Zone Northwest Ethiopia, 2019.

Of the total study participants, 512 (62.1%; 95% CI: 58.9%–65.3%) participants attended on-time appointments. Among the study participants who missed scheduled appointments, 159 (50.8%) and 87 (27.8%) missed the appointments due to forgetfulness and hopelessness, respectively (Figure 1).

### 3.5. Factors Associated with On-Time Appointment Keeping

Females have an increased odds of 1.49 (AOR = 1.49; 95% CI: 1.19–2.73) of keeping an appointment on-time compared to males. Age >24 years old had an increased odds of 2.13 (AOR = 2.13; 95% CI: 1.14–4.25) of keeping an appointment on-time compared to patients whose ages were between 18 and 24 years. Patients whose marital status was unmarried had almost 41% (AOR = 0.59; 95% CI: 0.45–0.82) decreased odds of keeping on-time appointments compared to those who were married. Those patients who have been taking the drug regimen of TDF + 3TC + EFV had an increased odds of 2.11 (AOR = 2.11; 95% CI: 1.84–3.62) of keeping on-time appointments as compared to patients who have been taking the drug regimen of others (AZT + 3TC + EFV, AZT + 3TC + NVP, or TDF + 3TC + NVP). HIV-positive patients who have been taking ART drugs for ≥12 years had an increased odds of 4.32 (AOR = 4.32; 95% CI: 2.22–8.40) of keeping on-time appointments as compared to patients who have been taking ART drugs for less than 12 months. Those patients who had mobiles had an increased odds of 2.29 (AOR = 2.29; 95% CI: 1.44–3.64) of keeping on-time appointments as compared to those who did not have mobiles. Patients who have gotten adherence support from peer support groups had an increased odds of 1.83 (AOR = 1.83; 95% CI: 1.21–3.34) of keeping on-time appointments compared to those patients who have not gotten adherence support from peer support groups. Those patients who have disclosed their HIV-positive status to their family members had an increased odds of 1.79 (AOR = 1.79; 95% CI: 1.14–3.23) of keeping an on-time appointment as compared to patients who have not disclosed their HIV-positive status to their family members. Patients who had paper-based appointments had an increased odds of 2.16 (AOR = 2.16; 95% CI: 1.16–3.50) of keeping on-time appointments as compared to those who did not have paper-based appointments (Table 4).

## 4. Discussion

The main objective of this study was to assess on-time appointment keeping and associated factors. Based on this objective, the prevalence of on-time appointment keeping was 62.1% (95% CI: 58.9%–65.3%). It is similar to the findings of the WHO Global Report from 50 countries (58%) [5].

This finding is lower than those of the studies conducted in East Africa, at 75.8% [19], Cambodia at 79.6% [20], Florida at 72.1% [21], and Malaysia at 81.3% [15]. Adult HIV patients often struggle with attendance at medical appointments due to various factors including work and childcare demands, lack of medical comorbidities, transportation issues, substance abuse, and untreated mental health conditions [22]. The discrepancy can be because of how HIV services are delivered differently. Currently, 95% of HIV services in Ethiopia are provided at facilities [23]. Contrarily, HIV services are delivered differently and in communities in other nations, which may have an impact on how often people keep their appointments.

The study found that factors such as sex, age, marital status, ART regimen, adherence support, family member disclosure, mobile access, and paper-based appointments significantly contribute to on-time appointment keeping in HIV treatment.

Females had increased odds of 1.49 of keeping an appointment on time compared to males. This is consistent with the findings of a study conducted in Uganda [24]. This might be due to the fact that men are more likely to use psychoactive substances such as alcohol, leading to depression and forgetting appointment times than women [25].

Another independent factor for on-time appointment keeping was age. Patients who were older than 24 years had almost fourfold higher odds of on-time appointment keeping than those who were aged between 18 and 24 years. This finding is congruent with that of the study conducted in Uganda [24]. The possible justification is that older age groups have a greater perception of health, or patients who are younger have different lifestyles (i.e., work or school status) that prevent them from maintaining consistent medical appointment attendance [21].

Patients who were unmarried had decreased odds of 41% of keeping on-time appointments compared to patients who were married. This could be because widowed patients were unable to receive partner support (emotional support) from their partners [24, 26, 27] or because they felt hopeless [28], both of which could have caused them to miss visits.

Patients who were taking the drug regimen of TDF + 3TC + EFV had an almost threefold increase in odds of on-time appointment keeping compared to those who were taking the regimen of another drug (AZT + 3TC + EFV, AZT + 3TC + NVP, or TDF + 3TC + NVP). A possible justification might be the presence of side effects. Patients may experience side effects such as nausea, dizziness, or fatigue, which can make it difficult for them to attend their health facility appointments regularly. In addition, the high pill burden associated with these regimens may lead to nonadherence and missed appointments [14, 29]. HIV-positive patients who were taking ART drugs for ≥12 months had fourfold higher odds of keeping on-time appointments than those patients who were taking ART drugs for less than 12 months. Patients who have taken ART drugs for <12 months may have adverse drug events, existing coinfections and/or comorbidities, severely low hemoglobin, a low body mass index (severe malnutrition), and/or very low CD4 counts [14, 29], which may cause them to miss the scheduled appointment.

Patients with mobiles had twofold higher odds of keeping on-time appointments than those without mobiles. This might be because patients who have mobile devices could receive a phone call or a reminder message from the healthcare providers. Mobile phone calling or messaging as a reminder improves adherence and clinic attendance [30].

Concerning adherence support from peer support groups, patients who have gotten adherence support from peer support groups had a more than 3-fold increase in the odds of on-time appointment keeping as compared to those patients who have not received adherence support from peer support groups. This is congruent with the findings of studies conducted in Uganda [31] and Malaysia [30]. This might be because patients with adherence support from peer support groups could get emotional support [24], which may impose on-time appointment keeping.

Furthermore, patients who disclosed their HIV-positive status to family members had a threefold increase in the odds of an on-time appointment keeping as compared with those who did not disclose their HIV-positive status. This might be because those patients who disclosed their HIV-positive status to their family members have gotten ART adherence and emotional support [24], which may enforce on-time appointment keeping.

Moreover, patients appointed with paper-based appointments experienced a sixfold increase in the odds of on-time appointment keeping as compared to those not appointed with paper-based appointments. This is in line with the finding of a study conducted in Africa [19]. A possible justification could be that patients who have appointments on paper will be less likely to forget [32].

### 4.1. Limitation of the Study

To avoid disclosure issues, data collectors were selected from the same health centers where the study participants were living. This could create an information bias.

## 5. Conclusion

The on-time appointment keeping was found to be lower than the WHO recommendations. Being female, being greater than 24 years old, being widowed, taking the regimen of TDF + 3TC + EFV, taking ART drugs for ≥12 months, having gotten adhesion support from peer support groups, disclosing HIV-positive status to a family member, having a mobile, and having paper-based appointments were factors that were significantly associated with on-time appointment keeping. Adherence support from family members, partners, and peer support groups, side effect management, and appointment reminders should be strengthened to increase on-time appointment keeping.

## Figures and Tables

**Table 1:** Sociodemographic and economic characteristics of HIV-positive patients accessing ART at health centers in East Gojjam Zone, Northwest Ethiopia, 2019.

Variables		Frequency	Percentage
Sex	Male	409	49.6
	Female	416	50.4

Age in year	18–24	30	3.6
	25–34	238	28.8
	35–44	372	45.1
	>44	185	22.4

Residence	Urban	469	56.8
	Rural	356	43.2

Educational status	No formal education	361	43.8
	Primary	279	33.8
	Secondary	158	19.2
	College and above	27	3.3

Occupation	Daily labourer	140	17.0
	Farmer	216	26.2
	Merchant	238	28.8
	Housewife	77	9.3
	Government employee	120	14.5
	Others	34	4.1

Marital status	Married	484	58.7
	Single	121	14.7
	Divorced	177	21.5
	Widowed	43	5.2

Average monthly income in birr	<500	35	4.2
	500–1000	84	10.2
	1001–1500	212	25.7
	1501–2000	154	18.7
	>2000	340	41.2

Mobile	Yes	649	78.7
	No	176	21.3

**Table 2:** Clinical characteristics of HIV-positive patients accessing ART at health centers in East Gojjam Zone, Northwest Ethiopia, 2019.

Variables		Frequency	Percentage
WHO clinical stage	Stage I	369	44.7
	Stage II	289	35.0
	Stage III	151	18.3
	Stage IV	16	1.9

CD4 count	<200	123	14.9
	200–500	354	42.9
	501–800	244	29.6
	>800	104	12.6

Functional status	Working	694	84.1
	Ambulatory	91	11.0
	Bedridden	40	4.8

Nutritional status	Normal	713	86.4
	Underweight	104	12.6
	Overweight	8	1.0

Drug regimen	TDF + 3TC + EFV	475	57.6
	Zidovudine AZT + 3TC + EFV	200	24.2
	AZT + 3TC + NVP (nevirapine)	80	9.7
	TDF + 3TC + NVP	51	6.2
	Abacavir (ABC) + 3TC + EFV	19	2.3

Encountered opportunistic infection	Yes	533	64.6
	No	292	35.4

Duration of treatment in a month	<12	70	8.5
	12–24	302	36.6
	25–36	289	35.0
	>36	164	19.9

Side effect	Yes	176	21.3
	No	649	78.7

**Table 3:** Psychosocial, behavioral, and healthcare delivery system characteristics of HIV-positive patients accessing ART at health centers in East Gojjam Zone, Northwest Ethiopia, 2019.

Variables		Frequency	Percentage
Perceived stigma	Yes	288	34.9
	No	537	65.1

Member association of PLWHA	Yes	495	60.0
	No	330	40.0

Social support	Yes	213	25.8
	No	612	74.2

Paper-based appointment	Yes	518	62.8
	No	307	37.2

Follow-up counseling	Yes	347	42.1
	No	478	57.9

Unavailability of health workers	Yes	219	26.5
	No	606	73.5

Appointment time convenient	Yes	336	40.7
	No	489	59.3

Substance abuse	Yes	398	48.2
	No	427	51.8

Satisfaction	Yes	329	39.9
	No	496	60.1

**Table 4:** Bivariable and multivariable logistic regression analysis of factors associated with on-time appointment keeping among HIV/AIDS-positive patients accessing ART at health centers in East Gojjam Zone, Northwest Ethiopia, 2019.

Variables	On-time appointment keeping		COR (95%) CI	AOR (95%) CI	*P* value
	Yes	No			
*Sex*					
Male	232	177	1	1	
Female	280	136	1.57 (1.18–2.08)	1.49 (1.19–2.73)	<0.001
*Age in years*	499	296			
18–24	13	17	1	1	
>24	499	296	2.20 (1.06–4.60)	2.13 (1.14–4.25)	0.003
*Marital status*					
Married	320	164	1	1	
Unmarried^*∗*^	192	149	0.66 (0.45–1.02)	0.59 (0.45–0.82)	0.001
*Drug regimen*					
TDF + 3TC + EFV	331	144	2.15 (1.61–2.86)	2.11 (1.84–3.62)	<0.001
Other^*∗∗*^	181	169	1	1	
*Duration of ART*					
<12 months	15	55	1	1	
≥12	497	256	7.06 (3.91–12.75)	4.32 (2.22–8.40)	<0.001
*Mobile*					
No	135	41	1	1	
Yes	377	272	2.38 (1.62–3.48)	2.22 (1.44–3.64)	<0.001
*Adherence support from peer support groups*					
Yes	154	59	1.85 (1.32–2.60)	1.83 (1.21–3.34)	<0.001
No	358	254	1	1	
*Disclosed to a family member*					
Yes	247	100	1.98 (1.48–2.66)	1.79 (1.14–3.23)	<0.001
No	265	213	1	1	
*Paper-based appointment*					
Yes	368	150	2.78 (2.07–3.72)	2.16 (1.16–3.50)	<0.001
No	144	163	1	1	
*Unavailability of health workers*					
Yes	148	71	1.39 (1.00–1.92)	1.15 (0.95–2.25)	0.088
No	364	242	1		
*Substance abuse*					
Yes	227	171	1	1	
No	285	142	0.66 (0.5–0.88)	0.56 (0.37–1.29)	0.466
*Satisfaction*					
No	298	198	1	1	
Yes	214	115	1.24 (0.93–1.65	1.15 (0.86–1.82)	0.233

^*∗*^Unmarried (single, divorced, and widowed). ^*∗∗*^AZT + 3TC + EFV, AZT + 3TC + NVP, TDF + 3TC + NVP, and ABC + 3TC + EFV.

## Data Availability

The corresponding author can provide the data that were used to support the findings of the study upon request.

## Abbreviations

3TC: Lamivudine
ABC: Abacavir
AIDS: Acquired immune deficiency syndrome
AOR: Adjusted odds ratio
ART: Antiretroviral therapy
AZT: Zidovudine
CD4: Cluster of differentiation 4
COR: Crude odds ratio
EFV: Efavirenz
HIV: Human immunodeficiency virus
IQR: Interquartile range
NVP: Nevirapine
TDF: Tenofovir
SPSS: Statistical Package for the Social Sciences
WHO: World Health Organization.

## Supplementary Materials

Supplementary Materials Appendices: data collection tools. Appendix 1: information and consent form. Appendix 2: checklist. Appendix 3: questionnaires.

## References

[1] World Health Organization (2004). Adherence to long term therapies–evidence for action. http://www.who.int/chp/knowledge/publications/adherence_full_report.pdf.

[2] World Health Organization (2016). *Report on Early Warning Indicators of HIV Drug Resistance*.

[3] Christian Obirikorang P. K. S., Abledu J. K., Opoku Fofie C. (2013). Predictors of adherence to antiretroviral therapy among hiv/aids patients in the upper west region of ghana. *ISRN AIDS*.

[4] Anoje C., Agu K. A., Oladele E. A. (2017). Adherence to on-time ART drug pick-up and its association with CD4 changes and clinical outcomes amongst HIV infected adults on first-line antiretroviral therapy in Nigerian hospitals. *AIDS and Behavior*.

[5] Bennett D. E., Jordan M. R., Bertagnolio S. (2012). HIV drug resistance early warning indicators in cohorts of individuals starting antiretroviral therapy between 2004 and 2009: world Health Organization global report from 50 countries. *Clinical Infectious Diseases*.

[6] Blacher R. J., Muiruri P., Njobvu L. (2010). How late is too late? Timeliness to scheduled visits as an antiretroviral therapy adherence measure in Nairobi, Kenya and Lusaka, Zambia. *AIDS Care*.

[7] Miller C. M., Ketlhapile M., Rybasack-Smith H., Rosen S. (2010). Why are antiretroviral treatment patients lost to follow-up? A qualitative study from South Africa. *Tropical Medicine and International Health*.

[8] Rosen S., Fox M. P., Gill C. J. (2007). Patient retention in antiretroviral therapy programs in sub-Saharan Africa: a systematic review. *PLoS Medicine*.

[9] Bofill L., Waldrop-Valverde D., Metsch L., Pereyra M., Kolber M. A. (2011). Demographic and psychosocial factors associated with appointment attendance among HIV-positive outpatients. *AIDS Care*.

[10] Israelski D., Gore-Felton C., Power R., Wood M. J., Koopman C. (2001). Sociodemographic characteristics associated with medical appointment adherence among HIV-seropositive patients seeking treatment in a county outpatient facility. *Preventive Medicine*.

[11] McGuire M., Pedrono G., Mukhua B., Huckabee M., Heinzelmann A., Szumilin E. (2011). Optimizing client monitoring after the first year of art: three years of implementing 6-monthly clinical appointments in rural malawi. *Abstract for IAS*.

[12] (2018). *East Gojjam Health Department 2018 Annual Report*.

[13] World Health Organization (2016). *Consolidated Guidelines on the Use of Antiretroviral Drugs for Treating and Preventing HIV Infection: Recommendations for a Public Health Approach*.

[14] Kebede W. (2017). *National Guidelines for Comprehensive HIV Prevention, Care and Treatment*.

[15] Abdulrahman S. A., Rampal L., Norlijah O., Ibrahim F., Kadir Shahar H., Radhakrishnan A. P. (2017). Sociodemographic profile and predictors of outpatient clinic attendance among HIV-positive patients initiating antiretroviral therapy in Selangor, Malaysia. *Patient Preference and Adherence*.

[16] Sangeda R. Z., Mosha F., Prosperi M. (2014). Pharmacy refill adherence outperforms self-reported methods in predicting HIV therapy outcome in resource-limited settings. *BMC Public Health*.

[17] Nikus Fido N., Aman M., Brihnu Z. (2016). HIV stigma and associated factors among antiretroviral treatment clients in Jimma town, Southwest Ethiopia. *HIV/AIDS-Research and Palliative Care*.

[18] Woodcock A., Bradley C. (2001). Validation of the HIV treatment satisfaction questionnaire (HIVTSQ). *Quality of Life Research*.

[19] Chalker J. C., Andualem T., Gitau L. N. (2010). Measuring adherence to antiretroviral treatment in resource-poor settings: the feasibility of collecting routine data for key indicators. *BMC Health Services Research*.

[20] Daigle G. T., Jolly P. E., Chamot E. A. (2015). System-level factors as predictors of adherence to clinical appointment schedules in antiretroviral therapy in Cambodia. *AIDS Care*.

[21] Bofill L. (2010). Demographic and psychosocial factors associated with appointment attendance among HIV positive outpatients. *International Journal of Infectious Diseases*.

[22] O’Connell K. A., Sherani S., Kisteneff A. (2022). Factors affecting adherence with follow-up appointments in HIV patients. *Cureus*.

[23] Assefa Y., Gilks C. F., Dean J. (2019). Towards achieving the fast-track targets and ending the epidemic of HIV/AIDS in Ethiopia: successes and challenges. *International Journal of Infectious Diseases*.

[24] Kunutsor S., Walley J., Katabira E. (2010). Clinic attendance for medication refills and medication adherence amongst an antiretroviral treatment cohort in Uganda: a prospective study. *AIDS research and treatment*.

[25] Center for Behavioral Health Statistics and Quality (2017). *Results from the 2016 National Survey on Drug Use and Health: Detailed Tables*.

[26] Hardon A., Davey S., Gerrits T. (2006). *From Access to Adherence: The Challenges of Antiretroviral Treatment: Studies from Botswana, Tanzania and Uganda 2006*.

[27] Asefa T., Taha M., Dejene T., Dube L. (2013). Determinants of defaulting from antiretroviral therapy treatment in nekemte hospital, eastern wollega Zone, western Ethiopia. *Public Health Research*.

[28] Forsythe S. S. (1998). The affordability of antiretroviral therapy in developing countries: what policymakers need to know. *Acquired Immune Deficiency Syndrome*.

[29] Tariku M. K., Worede D. T., Belete A. H. (2023). Adherence to antiretroviral therapy and associated factors among human immunodeficiency-positive patients accessing treatment at health centers in East Gojjam Zone, Northwest Ethiopia, 2019: community-based cross-sectional study. *Heliyon*.

[30] Abdulrahman S. A., Rampal L., Ibrahim F., Radhakrishnan A. P., Kadir Shahar H., Othman N. (2017). Mobile phone reminders and peer counseling improve adherence and treatment outcomes of patients on ART in Malaysia: a randomized clinical trial. *PLoS One*.

[31] Kunutsor S., Walley J., Katabira E. (2011). Improving clinic attendance and adherence to antiretroviral therapy through a treatment supporter intervention in Uganda: a randomized controlled trial. *AIDS and Behavior*.

[32] Shet A., De Costa A., Heylen E., Shastri S., Chandy S., Ekstrand M. (2011). High rates of adherence and treatment success in a public and public-private HIV clinic in India: potential benefits of standardized national care delivery systems. *BMC Health Services Research*.

