# Supplementary material for: On-Time Appointment Keeping and Associated Factors among Human Immunodeficiency Virus-Positive Adult Patients Accessing Antiretroviral Therapy at Health Centers in East Gojjam Zone, Northwest Ethiopia, 2019
Source: AIDS Res Treat. 2023 Nov 30;2023:1416187. doi: 10.1155/2023/1416187 (PMC10703533; doi:10.1155/2023/1416187)
Supplement: Supplementary Materials — Appendices: data collection tools. Appendix 1: information and consent form. Appendix 2: checklist. Appendix 3: questionnaires. [file 1416187.f1.docx]

**Appendices: Data collection tools**

**Appendix 1: Information and Consent form**

Bahir Dar university medicine and health science college department of Epidemiology and biostatistics

Questionnaire to assess On-time appointment keeping and associated factors among Human immunodeficiency virus-positive adult patients accessing antiretroviral therapy at Health centers in East Gojjam Zone, Northwest Ethiopia, 2019

Good morning/after noon

My name is-------------------------------- I am working as data collector in this study that to assess On-time appointment keeping and associated factors among Human immunodeficiency virus-positive adult patients accessing antiretroviral therapy at Health centers in East Gojjam Zone, Northwest Ethiopia, 2019 for an investigator doing his thesis for the partial fulfillment of Master’s degree in Field Epidemiology at Bahir Dar University, Ethiopia. You are selected by chance. Participation in this survey is voluntary and you can choose not to take part. However, your willingness to answer all of the questions is very important to you and other HIV/ADIS patients. Your name will not be written in this form and the information you will give to us is kept confidential. There will be no injection, drawing of blood or any blood fluid involved. You will be interviewed. It will not take more than 20 minutes. Generally, all information you will give will be used for only research purpose.

Are you voluntary to participate in this study?

A / yes B/ no

If the answer is yes, Thanks! Conduct

If the answer is no, Thanks! Transfer to the next respondent

Date of interview_________

**Appendix 2: Checklist**

Unique ART number------------

Sex ------------------

How long have he/she been on treatment for HIV/AIDS (ART) in month ---------------?

| S. N | Questions | Baseline | Most resent |
| --- | --- | --- | --- |
| 1 | Body weight in kg | ------------------- | ------------------------ |
| 2 | Body mass index in kg/m^2^ | -------------------- | ------------------- |
| 3 | CD4 cell count | ------------------- | --------------- |
| 4 | WHO clinical stage/Treatment stage | -------------- | ---------------- |
| 5 | Is Hemoglobin measured? | 1. Yes 2. No | 1. Yes 2. No |
| 6 | If Q5 is yes, what was the Status of anemia? | 1. Anemic 2. Non-anemic | 1. Anemic 2. Non-anemic |
| 7 | Has ever Viral load measured? | 1. Yes 2. no | 1. Yes 2. No |
| 8 | If Q7 is yes, how much copies/m? | ------------ | -------------- |
| 9 | What was the baseline drug regimen? | | |
| 10 | Is the baseline drug regimen changed? 1. Yes 2. No | | |
| 11 | If Q10 is yes, to what regimen? | | |
| 12 | Is the appointment kept on time? 1. Yes 2. No | | |
| 13 | Had ever history of taking IPT? 1. Yes 2. No | | |
| 14 | Had history of taking Cotrimoxazole Preventive Therapy? 1. Yes 2. No | | |
| 15 | Had history of Opportunistic infection? 1. Yes 2. No | | |
| 16 | Had history of Tuberculosis? 1. Yes 2. No | | |
| 17 | If Q16 is yes what type of TB? 1. Extra PTB 2. PTB positive 3. PTB negative | | |
| 18 | Resent partner HIV status 1. Known 2. Unknown | | |
| 19 | If q15 is 1 what is the result 1. Negative 2. Positive | | |
| 20 | Is there a reported side effect? 1. Yes 2. No | | |
| 21 | Baseline Functional status1. Working 2. Ambulatory 3. Bedridden | | |
| 22 | Is appointment date registered? 1. Yes 2. No | | |

**Appendix 3: Questionnaires**

Unique ART number------------

**Instruction**- circle the response for question with alternative and write for open ended question on the space provide

| **Part I: Socio demographic characteristics of study participants** | | | |
| --- | --- | --- | --- |
| S. No. | Question | Answer and Code | Skip |
| 101 | Age in year | ----------------- |  |
| 102 | Residence | 1. Urban 2. Rural |  |
| 103 | Level of education | 1. No formal education 2. Primary 3. Secondary 4. College and above |  |
| 104 | Occupation | 1. Daily laborer 2. Farmer 3. merchant 4. governmental Employee 5. Housewife 6. Others |  |
| 105 | Current Marital status | 1. Never Married 2. Married and live together 3. Married and live in Separated 4. Divorced 5. Widowed |  |
| 106 | Religion | 1. Orthodox 2. Muslim 3. Protestant 4. Catholic 5. Others |  |
| 107 | Do you have a child? | 1. Yes 2. No | If the answer is no, skip to Q109 |
| 108 | If Q107 is yes, how many? | ------------ |  |
| 109 | Number of family member in the household | ----------- |  |
| 110 | Monthly income in Ethiopian birr | -------- |  |
| 111 | Is there a family member co enrollment in ART? | 1. Yes 2. No |  |

| **Part II: Patient satisfaction with ART services** | | | | | | |
| --- | --- | --- | --- | --- | --- | --- |
| **S. No.** | **Question** | **Alternatives** | | | | |
|  |  | Strongly disagree | Disagree | Uncertain | Agree | Strongly agree |
| 201 | Has an excellent professional relationship with you |  |  |  |  |  |
| 202 | Spends as much time as you need with you |  |  |  |  |  |
| 203 | Is always available and answers your questions well |  |  |  |  |  |
| 204 | You are shown courtesy and respect by the pharmacy staff |  |  |  |  |  |
| 205 | Constantly emphasizes on the importance of taking your medications as prescribed  (Adherence) |  |  |  |  |  |
| 206 | Gives you information about the results to expect from your drug therapy |  |  |  |  |  |
| 207 | Advises you about problems that might occur with your medications (side effects) |  |  |  |  |  |
| 208 | Advises you on the types of food you should eat (nutrition) |  |  |  |  |  |
| 209 | Gives you information on some drugs and other things to avoid while on your  medications |  |  |  |  |  |
| 210 | Always determines how much knowledge/information you have about the HIV  disease and your medications |  |  |  |  |  |
| 211 | Informs you of the purpose of your medications |  |  |  |  |  |
| 212 | Provides you with written information about your drugs and disease |  |  |  |  |  |
| 213 | Ask questions about your previous illnesses and medications taken |  |  |  |  |  |
| 214 | Works together with you to choose a medication schedule that is most convenient  for you |  |  |  |  |  |
| 215 | Always seeks to know if you have any health problems related to your medications |  |  |  |  |  |
| 216 | Renders sufficient help when you have problems related to your medications |  |  |  |  |  |

| **Part III: Psycho-social characteristics** | | | |
| --- | --- | --- | --- |
| **S. No.** | **Question** | **Answer and Code** | **Skip** |
| 301 | Are you member of the Association of PLWHA? | 1. Yes 2. No |  |
| 302 | Have you ever gote social support? | 1. Yes 2. No |  |
| 303 | Have ever you disclosed your status? | 1. Yes 2. No | If the answer is no, skip to Next part |
| 304 | If Q303 is yes, to whom? | 1. To my partner 2. My father 3. My mother 4. My children 5. Friends 6. Others |  |

**Perceived social stigma**

| **Questions** | **Yes(1)** | **No(0)** |
| --- | --- | --- |
| I am afraid about people discriminating against me |  |  |
| I am worried that people may tell my HIV status to others |  |  |
| I told people close to me to keep my HIV status as a secret |  |  |
| I think that people would be afraid of me if they knew my HIV status |  |  |
| I am working hard to keep my HIV status as a secret |  |  |
| I feel that no one knows my HIV-positive status |  |  |
| I feel guilty because of my HIV-positive status |  |  |
| I feel I am a bad person due to my HIV-positive status |  |  |
| People told me that HIV is what I deserved |  |  |
| I feels I am unclean due to my HIV-positive status |  |  |
| I decided not to attend social gatherings due to HIV-positive status |  |  |
| I decided to withdraw from education/training |  |  |

| **Part IV: Health System Factors** | | | |
| --- | --- | --- | --- |
| **S. No.** | **Question** | **Answer and Code** | **Skip** |
| 401 | Have you ever got pre- ART counseling? | 1. Yes 2. No | If the answer is no, skip to Q403 |
| 402 | If Q401 is yes, what is your satisfaction? | 1. More satisfied 2. Moderately satisfied 3. Satisfied 4. Not satisfied |  |
| 403 | Waiting time (in munities) of the recent visit at clinics | -------------------- |  |
| 404 | Have you ever faced unavailability of health providers during visits? | 1. Yes 2. No |  |
| 405 | Have you ever faced unavailability of drug at health center during visits? | 1. Yes 2. No |  |
| 406 | Is the appointment time’s convenience? | 1. Yes 2. No |  |

| **Part V: Other Factors** | | | |
| --- | --- | --- | --- |
| **S. No.** | **Question** | **Answer and Code** | **Skip** |
| 501 | Have you ever used traditional medicine? | 1. Yes 2. No |  |
| 502 | What is your mode of travel to ART health center? | 1. By car 2. On foot 3. Others | If the answer is 1 and 3 skips to 505 |
| 503 | If Q503 is on foot, average time taken to reach health center | 1. Less than one hour 2. One hour 3. Greater than one hours |  |
| 504 | Do you have telephone? | 1. Yes 2. No |  |
| 505 | Functional status | 1. Working 2. Ambulatory 3. Bedridden |  |
| 506 | What is the reason for missing the appointment? | 1. Forgetfulness 2. Hopelessness 3. Lack of transport money 4. Side effect 5. Others (specify) |  |
| 507 | After you have been on ART, have you ever smoking? | 1. Yes 2. No | If the answer is 2 skips to 509 |
| 508 | If Q507 is yes How often | 1. Daily 2. Occasionally |  |
| 509 | After you have been on ART, have you ever drinking alcohol | 1. Yes 2. No | If the answer is 2 skips to 511 |
| 510 | If Q509 is yes, How often | 1. Daily 2. Occasionally |  |
| 511 | After you have been on ART, have you ever chewing Khat? | 1. Yes 2. No | If the answer is 2 say thank and stop |
| 512 | If Q511 is yes, How often | 1. Daily 2. Occasionally |  |
